# Introduction of the Ex/AxI ratio for optimized assessment of exophthalmos based on bulbar position parameters examined in a population-based MRI study

**DOI:** 10.1038/s41598-026-35424-6

**Published:** 2026-01-16

**Authors:** Lisa Lüdtke, Till Ittermann, Clemens Jürgens, Sönke Langner, Achim Beule, Henry Völzke, Andreas Stahl, Frank Tost

**Affiliations:** 1 Department of Ophthalmology, University Medicine Greifswald, Ferdinand-Sauerbruch-Strasse, 17475 Greifswald, Germany; 2 Institute for Community Medicine, University Medicine Greifswald, Greifswald, Germany; 3 Institute for Diagnostic Radiology and Neuroradiology, University Medicine Greifswald, Greifswald, Germany; 4 Clinic for Ear, Nose, and Throat Disorders, University Hospital Münster, Münster, Germany

**Keywords:** Exophthalmometry, Protrusio bulbi, Exophthalmos, Quotient Ex/AxI, Anthropometry, Magnetic resonance imaging, Diseases, Health care, Medical research

## Abstract

Exophthalmometry is an essential component in the determination of changes in the position of the eyeball and is influenced by various factors. This paper analyzes the usefulness of the Ex/Axl ratio, meaning the ratio of anterior exophthalmos to bulbar axial length, in relation to the position of the eye in the orbit in order to take better account the anthropometric characteristics of individual exophthalmometry measurements. MRI scans of the head in the population-based cohorts SHIP-START-2 and SHIP-TREND-0 were used to perform anterior and posterior exophthalmometry as well as bulbar axial length measurements in 2083 subjects between 21 and 89 years of age. After excluding non-measurable images, 1926 subjects were included in the data analysis. We established sex- and age specific reference limits for the Ex/Axl ratio and the exophthalmometry parameters. If the Ex/Axl ratio was above 85.3% in men and above 79.7% in women, this indicated pathological exophthalmos in our study. Exophthalmometry should be considered on a sex-specific basis since there are clear differences in the measurements between men and women. MRI images of the head were used to determine separate reference values for men and women, which may be useful in clinical practice. Furthermore, our study highlights the clinical significance of measuring the individual bulbar axial length and anthropometric data for the personalized assessment of exophthalmos. In this context, the newly introduced Ex/Axl ratio may be a useful tool for a better differentiation between pseudoexophthalmos and clinically relevant exophthalmos.

## Introduction

Exophthalmometry is an essential part of the examination of changes in the position of the eye. Both systemic diseases and localized pathological changes in the orbit and its surrounding tissue should be considered. In adults, these changes primarily include endocrine orbitopathy in the context of Graves’ disease. In children, unilateral exophthalmos often indicates a sinugenic problem, while bilateral exophthalmos is more likely to indicate the presence of leukemia or neuroblastoma metastases^1^. In clinical practice, exophthalmos is usually determined non-invasively and efficiently using an exophthalmometer, e.g. according to Hertel^1^. A side difference of 2 mm is regarded as clearly pathological and requires further medical diagnostic clarification^1^. It should be noted that with exophthalmometry, only the position of parts of the anterior segment of the eye is used for measurement. A more detailed clinical workup requires multimodal imaging to also assess intraorbital, retrobulbar and ocular structures.

The position of the eyeball within the orbit depends on various physiological factors, such as the bone orbital structures and the orbital fat tissue^2^. Furthermore, ethnic origin plays a role in this context due to the different characteristics of skull morphology^3–6^. The length of the bulbar axis is also of practical importance for the assessment of extraorbital prominence, e.g. in cases of unilateral high myopia^5^. Anthropometric factors such as waist and hip circumference and body-mass index (BMI) also play a role^7^.

We therefore investigated the position of the eye in the orbit by assessing anterior and posterior exophthalmometry and bulbar axis length using magnetic resonance (MR) images of the head with the aim of testing a ratio for optimized differentiation between exophthalmos with disease value and pseudoexophthalmos using medical data from the population-based Studies of Health in Pomerania (SHIP-START-2 and SHIP-TREND-0).

## Methods

SHIP is a population-based project in northeastern Germany that investigates the determinants of health and disease and the interactions of risk factors, subclinical changes and population-relevant diseases^8^.

In the two independent cohorts SHIP-2 and SHIP-TREND-0, an optional whole-body MR using 1.5 T scanner Magnetom Avanto (Siemens Medical Solutions, Erlangen, Germany) was performed. The standardized examination protocol is available^8^. All subjects gave written informed consent. The study was approved by the local Ethics committee and follows the Declaration of Helsinki.

Of the total 6753 subjects included in both cohorts, 4121 subjects could not undergo an MRI due to existing exclusion criteria. These included metallic implants, tattoos or claustrophobia. A further 549 subjects showed limited image quality, so that ultimately 2083 subjects aged between 21 and 89 years were available for evaluation. The examination was carried out using transverse T1-weighted images of the head with a section thickness of 1 mm and a field of view of 256 × 256 mm. Further technical parameters are listed in Table 1. The images were examined and measured using the DICOM viewer OsiriX (Pixmeo, Geneva, Switzerland). The zoom was set to 750% and the contrast was defined in the W/L setting of OsiriX with the setting “0” to ensure comparability of the measurements. The apex of the cornea and the optic disc had to be visible in the same image plane. Accordingly, all image planes that did not meet this criterion were excluded. Further exclusion criteria according to the quality of the image planes were “only one eye could be examined” and “bulb length could be determined, but exophthalmometry was not possible”. After applying these additional exclusion criteria, a total of 1926 subjects were available for the final analysis.

**Table 1:** MRI acquisition parameters.

sequence	repetition time (ms)	echo time (ms)	flip angle (degree)	voxel size (mm)	time (min)
T1 MPR ax	1900	3.4	15	1.0 × 1.0x1.0	3:38

Legend: ax = axial, MPR = multiplanar reconstruction.

**Fig. 1:**
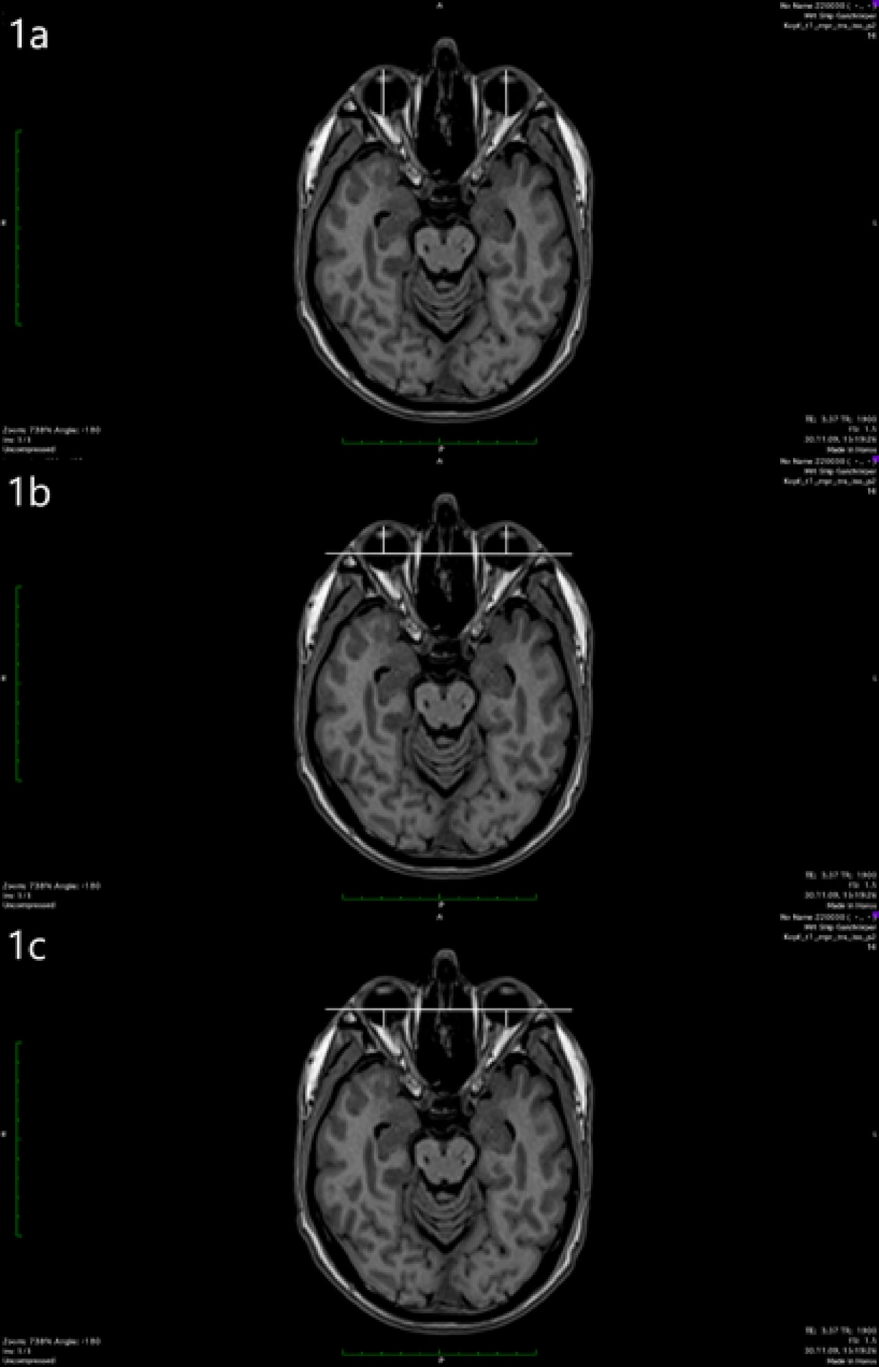
(**1a**) Bulbar axial length, (**1b**) Anterior exophthalmometry, (**1c**) Posterior exophthalmometry of the eye in an axial T1-weighted MRI.

**Fig. 2:**
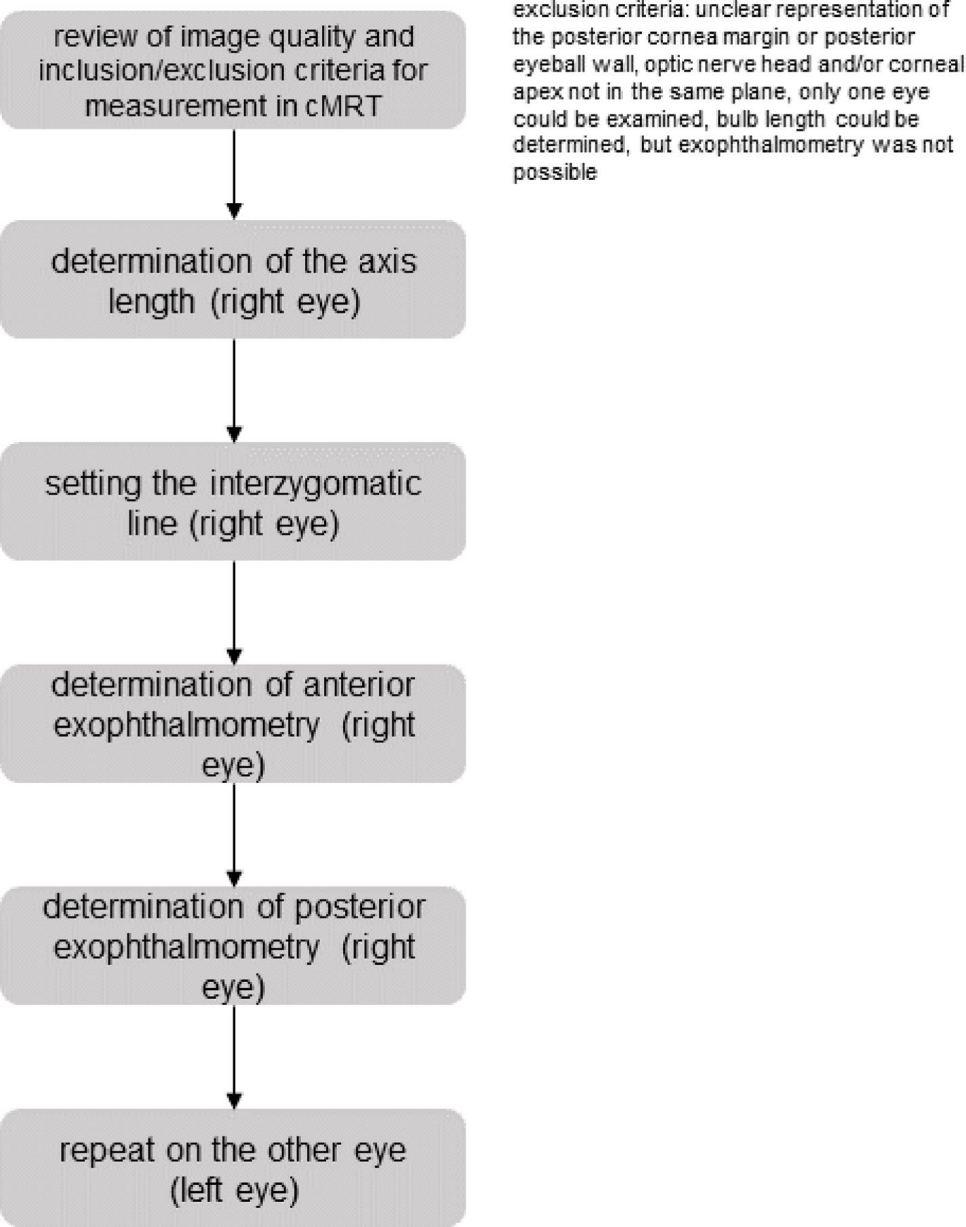
Flowchart: representation of the individual measurement steps of the MRI scans

The length of the eye ball was measured between the posterior surface of the cornea, which was better visible on MRI images than the anterior surface, and the posterior pole of the ocular globe (Fig. 1a). The measurement was repeated for several scans. The maximum value determined from all measurements corresponded to the bulbar axial length. The selected image plane was chosen for all further measurements on both eyes. For exophthalmometry, a connecting line was drawn between the lateral bony boundaries of the orbits. This line, also known as the interzygomatic line, lies on the bony orbital margins without intersecting them. Anterior exophthalmometry is measured from the posterior surface of the cornea at the level of the corneal apex, starting from the interzygomatic line (Fig. 1b). Posterior exophthalmometry is performed accordingly between the interzygomatic line and the posterior pole at a 90° angle (Fig. 1c). The corresponding flowchart illustrating the study design is shown in Fig. 2.

In order to introduce reference values for exophthalmometry in the general German population, a reference group is formed. As the position of the eye depends on various factors, the inclusion criteria were defined as follows:

- Age over 30 years of age
- Bulbar axial length less than 24 mm
- BMI below 30 kg/m^2^
- No thyroid disease (stated in the standardized interview)

The included subjects with corresponding anterior exophthalmometry measurementes are shown in Table 4.

**Fig. 3:**
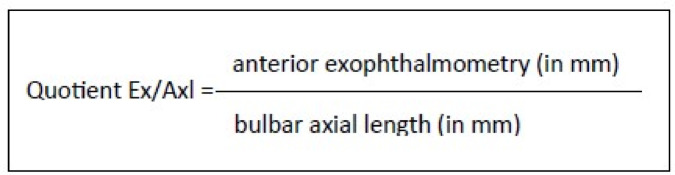
Quotient Ex/Axl. The quotient consists of the anterior exophthalmometry and the bulbar axial length together.

A ratio was formed from the measured anterior exophthalmometry and bulbar axial length (Fig. 3) to enable simple differentiation between pseudoexophthalmos with high bulbar axial length and clinically significant exophthalmos. The quotient ranges between 0 and 100%.

### Statistical analyses

Stratified by sex continuous data was expressed as median, 25th, and 75th percentile. Differences of the parameters between men and women were evaluated by Mann–Whitney U tests.

Multivariable analyses were conducted on basis of the examined eyes with each participant having two values one for the right and one for the left eye (*n* = 3,852 eyes). The potential risk factors age, BMI, height, weight, waist circumference, hip circumference, total cholesterol, LDL cholesterol, HDL cholesterol, triglycerides, HbA1c and TSH were associated with the Ex/Axl quotient by linear mixed models with random intercept adjusted for age and stratified by sex (Fig.5). The results are expressed as β coefficients with 95% confidence intervals. A p < 0.05 was considered as statistically significant. Since not all individuals conducted the MRI examinations, we calculated inverse probability weights for non-participation. These weights were applied to all regression analyses.

In the healthy reference population sex-specific reference limits were calculated by quantile regression models for the 95th percentile. Additionally, age was introduced in these regression models. Statistical analysis was performed using Stata 18.5 (STATA corp., College Station, TX, USA).

### Results

The average age of the subjects included in this study was 53 years with a proportion of 45% female participants. The analysis of intraindividual differences between the eyes showed no significant difference. However, all parameters measured showed a significant difference between men and women (Table 2). Posterior exophthalmometry values were higher in women than in men, whereas values of anterior exophthalmometry, and bulbar axial length were higher in men compared to women. A side difference of 2 mm in exophthalmometry is generally considered pathologic. In our data using anterior exophthalmometry, such a difference was found in a total of 20 subjects (1.0%). However, it should be noted that this classification refers to the examination with a hand-held exophthalmometer and that our measurements in the MRI images were based on the posterior edge of the cornea.

**Table 2:** Characteristics of the study population stratified by sex.

	Men	Women	p*
N	1,059 (55.0%)	867 (45.0%)	
Age in years	53 (42; 64)	54 (44; 64)	0.075
Height in cm	177 (172; 180)	164 (159; 168)	< 0.001
Weight in kg	86 (78; 96)	74 (64; 83)	< 0.001
Body mass index	27.8 (25.4; 30.4)	27.5 (24.0; 31.4)	0.082
Waist circumference in cm	95 (88; 103)	85 (77; 95)	< 0.001
Hip circumference in cm	101 (96; 105)	102 (95; 111)	< 0.001
Total cholesterol in mmol/L	5.40 (4.60; 6.10)	5.60 (4.90; 6.40)	< 0.001
LDL-cholesterol in mmol/L	3.41 (2.72; 3.96)	3.39 (2.79; 4.09)	0.315
HDL-cholesterol in mmol/L	1.25 (1.05; 1.48)	1.53 (1.31; 1.80)	< 0.001
Triglycerides in mmol/L	1.54 (1.06; 2.30)	1.31 (0.94; 1.83)	< 0.001
HbA1c in %	5.30 (5.00; 5.70)	5.30 (4.90; 5.60)	0.008
TSH in mIU/L	1.09 (0.73; 1.51)	1.09 (0.74; 1.64)	0.243
Anterior exophthalmometry in mm (right eye)	16.4 (15.1; 18.0)	15.3 (13.9; 16.8)	< 0.001
Anterior exophthalmometry in mm (left eye)	16.3 (15.0; 17.9)	15.2 (13.8; 16.6)	< 0.001
Posterior exophthalmometry in mm (right eye)	6.8 (5.3; 8.3)	7.3 (5.8; 8.8)	< 0.001
Posterior exophthalmometry in mm (left eye)	6.9 (5.3; 8.3)	7.5 (6.0; 8.9)	< 0.001
Bulbar axis length in mm (right eye)	23.4 (22.9; 23.9)	22.7 (22.2; 23.3)	< 0.001
Bulbar axis length in mm (left eye)	23.4 (22.8; 23.9)	22.7 (22.2; 23.3)	< 0.001
Ex/Axl ratio in % (right eye)	70.3 (64.5; 76.4)	67.5 (61.1; 73.4)	< 0.001
Ex/Axl ratio in % (left eye)	69.7 (64.4; 76.8)	66.5 (60.4; 72.7)	< 0.001

Data are expressed as Median, 25^th^ and 75^th^ percentile; N = number.

*Mann–Whitney-U test.

The following analyses were all conducted eye level with each participant having two values one for the right and one for the left eye (*n* = 3,852 eyes). We observed significant association of bulbar axial length with anterior and posterior exophthalmometry (*p* < 0.001). In a linear mixed-model adjusted for age and sex, each mm higher bulbar axial length was associated with a 0.20 mm higher anterior exophthalmometry and a 0.82 mm higher posterior exophthalmometry.

This correlation is taken into account in the introduction of the Ex/Axl ratio. The ratio is derived by measuring the anterior exophthalmometry and bulbar axial length. The Ex/Axl ratio was found to be significantly higher in men than in women (Table 4). Furthermore, we observed positive association of the Ex/Axl ratio with weight, BMI, waist circumference, hip circumference, triglycerides and HbA1c in men and women, whereas the Ex/Axl ratio was inversely associated with HDL-cholesterol in both sexes (Table 3). Only in women, we found a positive association between the Ex/Axl ratio and LDL cholesterol. There were no significant associations of the Ex/Axl ratio with total cholesterol or serum TSH levels .

**Table 3:** Sex-specific associations of potential risk factors with the Ex/Axl ratio on eye basis.

	Men β (95% confidence interval)	Women β (95% confidence interval)
Height in cm	− 0.06 (− 0.15; 0.04)	− 0.01 (− 0.11; 0.08)
Weight in kg	0.21 (0.16; 0.25)*	0.22 (0.18; 0.26)*
Body mass index	0.88 (0.73; 1.04)*	0.62 (0.51; 0.73)*
Waist circumference in cm	0.29 (0.24; 0.35)*	0.27 (0.22; 0.31)*
Hip circumference in cm	0.26 (0.19; 0.34)*	0.26 (0.21; 0.31)*
Total cholesterol in mmol/L	0.00 (− 0.51; 0.51)	0.56 (− 0.02; 1.14)
LDL-cholesterol in mmol/L	− 0.21 (− 0.81; 0.38)	0.86 (0.25; 1.48)*
HDL-cholesterol in mmol/L	− 3.72 (− 5.58; − 1.86)*	− 4.38 (− 6.05; − 2.70)*
Triglycerides in mmol/L	1.03 (0.60; 1.45)*	1.88 (1.04; 2.73)*
HbA1c in %	1.08 (0.25; 1.91)*	2.58 (1.56; 3.60)*
TSH in mIU/L	− 0.26 (− 0.87; 0.36)	0.27 (− 0.12; 0.67)

Linear mixed model with random intercept adjusted for age (except when age is exposure).

*p < 0.05.

**Fig. 4:**
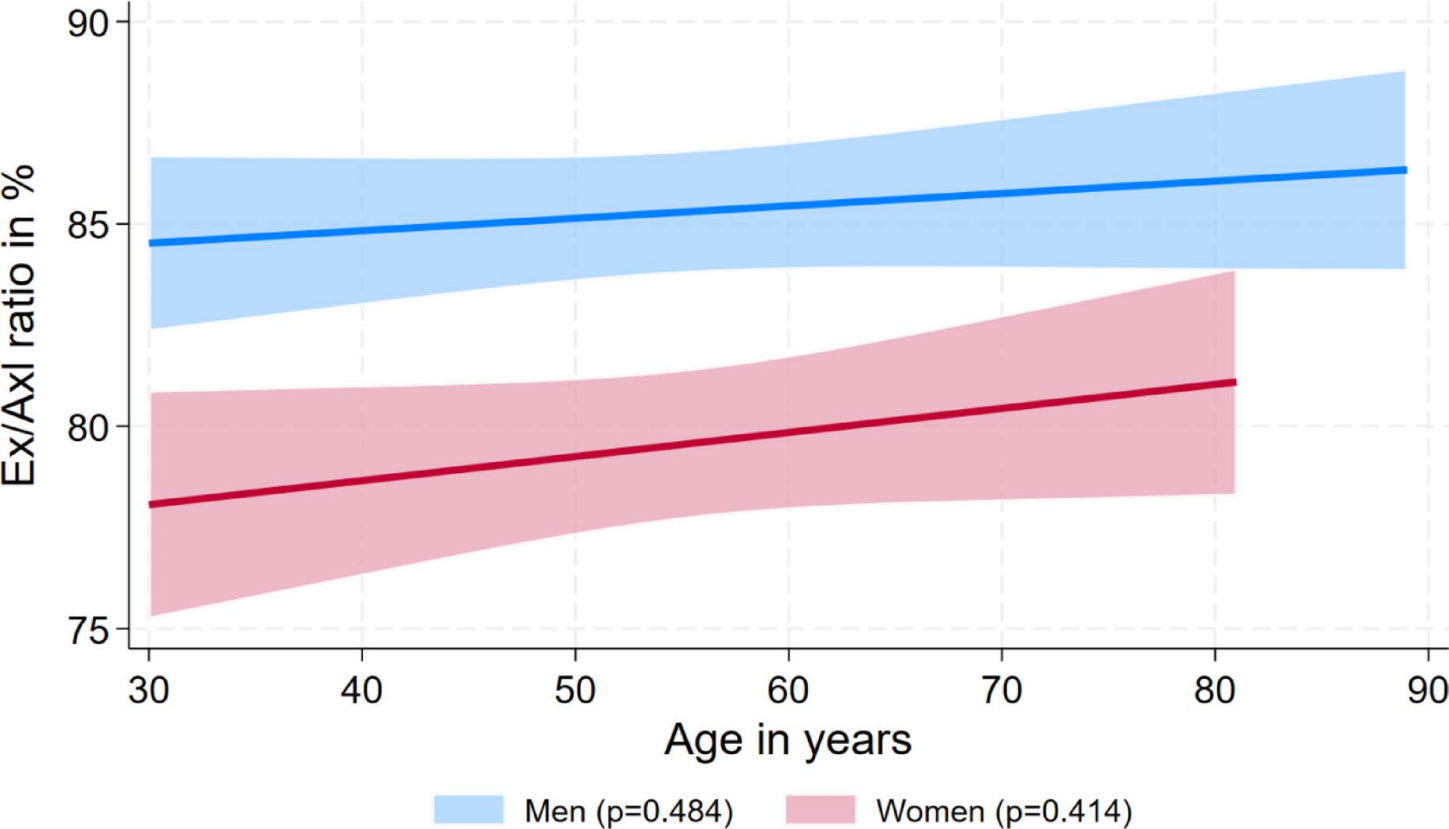
Sex-specific reference values (95th percentile) for the Ex/Axl quotient by age. The gender-specific analysis shows clear differences between men and women in terms of the Ex/Axl ratio.

**Fig. 5:**
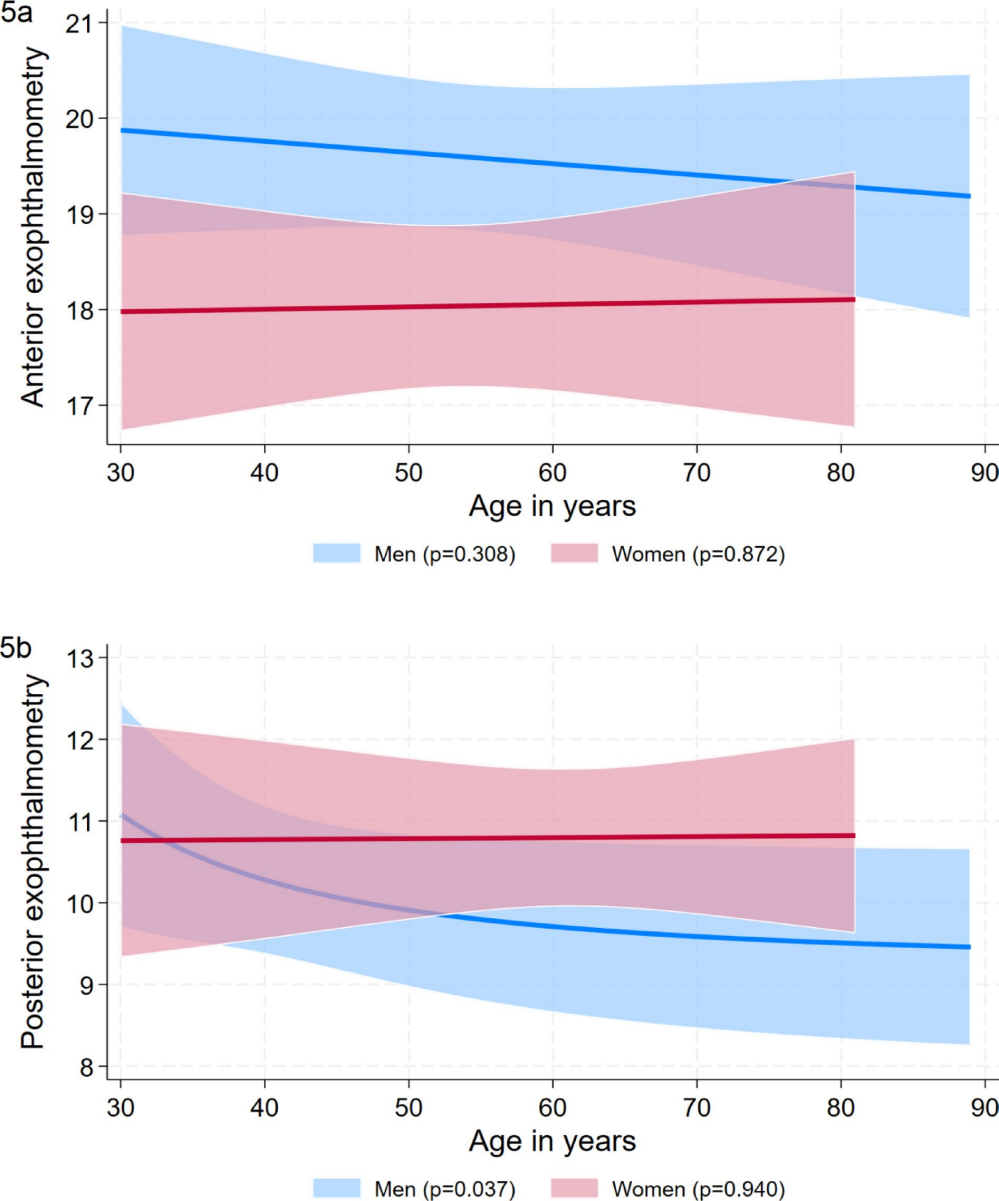

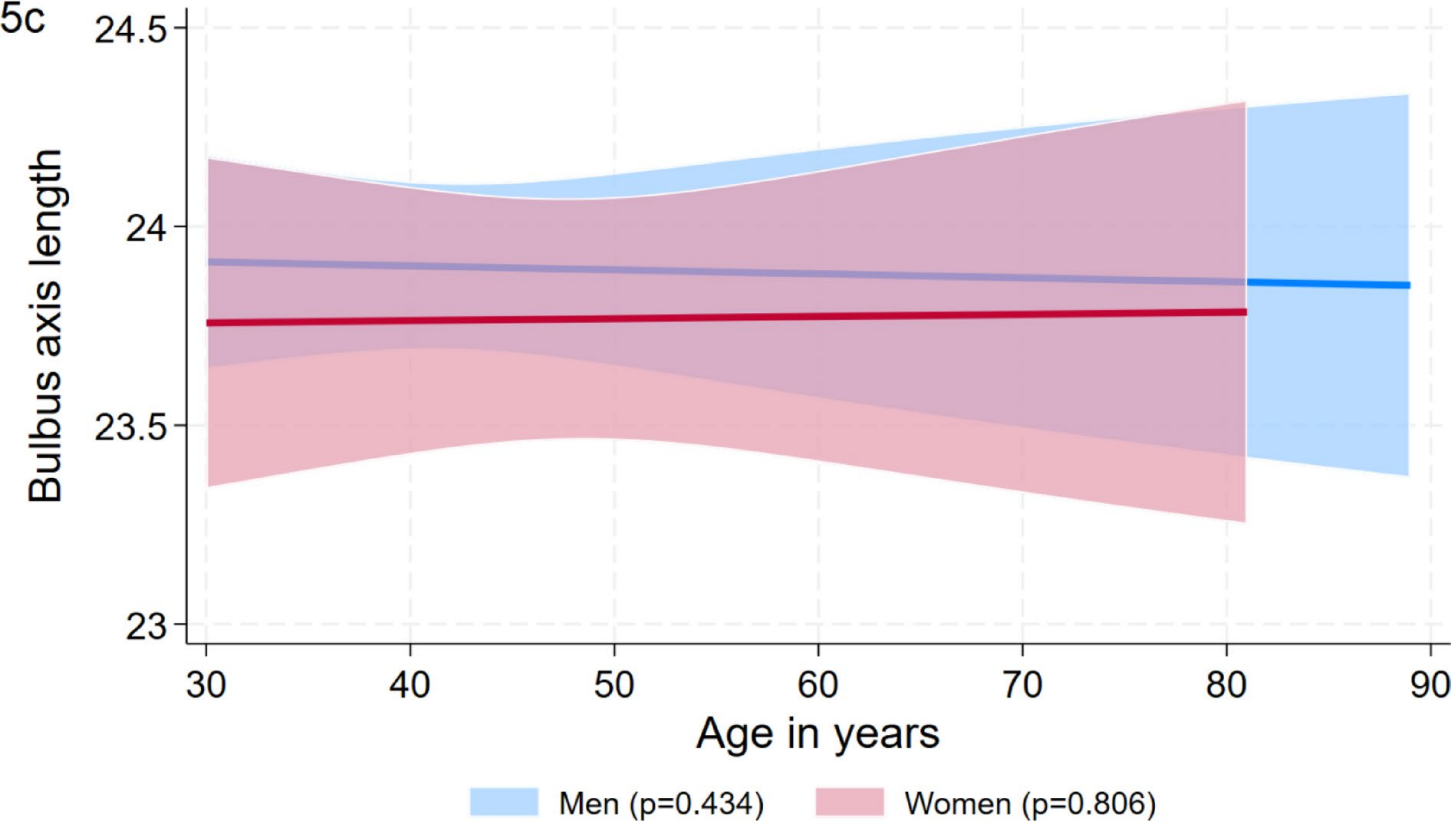
(a-c) Sex-specific reference values (95th percentile) for anterior exophthalmometry, posterior exophthalmometry and bulbar axis length percentile by age. When analysing the individual parameters of exophthalmometry, gender-specific differences can be seen, as in Fig. 4. While men showed higher values in the measurement of anterior exophthalmometry and bulbar axis length, higher values were determined for women in posterior exophthalmometry..

To determine a healthy reference population, we excluded 2,156 eyes from individuals with a BMI > 30 kg/m^2^, a self-reported thyroid disease, an age < 30 years, and a bulbar axial length > 24 mm resulting in 1,696 eyes. In this group, reference values for anterior exophtalmometry and Ex/Axl ratio were higher in men than in women, whereas reference values for posterior exophthalmometry were higher in women than in men and reference limits for bulbar axis length were comparable among the two sexes (Table 4). The overall reference values for the Ex/Axl ratio were 85.3% in men and 79.7% in women. Results above these values indicated a pathological exophthalmos in our study. Age-specific formulas for the reference limits of the exophthalmometric markers are shown in Table 5 and visualized in Figs. 4 and 5a–c. A significant association of age was only observed for the 95^th^ percentile of posterior exophthalmometry in men.

**Table 4:** Reference limits by sex based on the reference group for anterior exophthalmometry. posterior exophthalmometry, bulbar axis length, and the Ex/Axl ratio.

	Men	Women
Anterior exophthalmometry in mm	19.6 (19.3; 19.9)	18.1 (17.7; 18.4)
Posterior exophthalmometry in mm	10.0 (9.6; 10.3)	10.8 (10.4; 11.2)
Bulbar axis length in mm	23.9 (23.8; 23.9)	23.8 (23.7; 23.8)
Ex/Axl ratio in %	85.3 (84.1; 86.6)	79.7 (78.2; 81.3)

Results are based on quantile regression for the 95^th^ percentile and expressed as 95^th^ percentile with 95% confidence interval.

**Table 5:** Sex-specific formulas for the exophtalmometry parameters by age.

	Formula
Anterior exophthalmometry in mm	
Men	20.2269 – 0.01172*age
Women	17.9025 + 0.0025*age
Posterior exophthalmometry in mm	
Men	9.2511 + 1646*1/age^2
Women	10.7239 + 0.00121*age
Bulbar axial length	
Men	23.941 – 0.001*age
Women	23.7421 + 0.00053*age
Ex/Axl ratio in %	
Men	83.6014 + 0.03073*age
Women	76.2776 – 0.05946*age

## Discussion

In clinical practice, exophthalmos is most frequently determined using a hand-held exophthalmometer, because it can be performed quickly and easily with minimal stress on the patient. Imaging techniques are among the advanced diagnostic methods for protrusio bulbi. MRI plays a particularly important role in the assessment of Graves’ disease, as it provides visualization of changes in soft tissue structures, such as swelling of the eye muscles, and allows for assessment of disease activity during treatment^9^.

The work of Bingham et al. and Hauck et al. demonstrated good comparability between measurements using hand-held exophthalmometers and anterior exophthalmometry using cross-sectional computer tomographic imaging^10,11^. It should be noted that these measurements were taken from the anterior corneal surface. Segni et al., in contrast, performed the measurements using computed tomography (CT) scans from the posterior surface of the cornea and found lower values compared to hand-held exophthalmometry, which are comparable to the results of our study^12^. Since our study population consists of a cross-section of the general population, it can be assumed that the central corneal thickness is between 520 and 560 µm and therefore a correction factor of 0.5 mm facilitates comparability with other studies.

Overall, there are only a few studies on the position of the eye in healthy Caucasians. Our measurement results are comparable with the work of Lang et al. and Detorakis et al.^13,14^. Both studies were also able to demonstrate a difference in the position of the eye within the orbit between men and women, with women showing lower values of anterior and posterior exophthalmometry as well as shorter bulbar axial length. Our study differed from these reported results by noting slightly higher posterior exophthalmometry values in women compared to men.

In contrast, Mourtis et al. and Ahmadi et al. showed measured values that differed from our results. One possible reason for this could be that these studies were not population-based, significantly fewer subjects were examined and the data were not collected using MRI imaging^3,15^.

A reference group was established in our study in order to introduce reference values for exophthalmometry for the general German population. The inclusion criteria were age over 30 years, a bulbar axial length of less than 24 mm, BMI of less than 30 kg/m^2^ and the exclusion of thyroid disease. Chan et al. as well as the studies by Ahmadi et al. and Beden et al. considered all values with a standard deviation of + / − 1.96 for anterior exophthalmometry. When looking at our values in Table 4, a clear difference between men and women becomes apparent, so that it does seem sensible to set different reference values for men and women.

In an international comparison, an influence of ethnic origin on bulb position in the orbit becomes apparent. While study data from Mexico and Nigeria^16,17^ are on average comparable with our results, studies of the population in Sri Lanka^5^ and the African-American population in the USA^5^ show significantly higher values of anterior exophthalmometry. In contrast, lower values were documented for the population of Taiwan^18^ and Turkey^19^ using anterior exophthalmometry. The reason for the differences in measured values is likely to be found in the different characteristics of the skull morphology^3^. People of Afro-Caribbean origin have a flatter eye socket compared to Central Europeans. In addition, the protrusion of the eyeball is more pronounced in people of Afro-Caribbean origin. Therefore, ethnic origin should always be taken into account when assessing the position of the eyeball in the orbit.

The examination using posterior exophthalmometry in our study was, to our knowledge, performed for the first time on a large number of subjects. In our reference group, mean values of posterior exophthalmometry differed compared to anterior exophthalmometry measurements (Table 4). In addition, the data collected prior to our study was mainly collected on CT scans. For example, the work of Lee et al. and Sheik et al. was performed on CT scans^20,21^. While Lee et al. reported higher values in posterior exophthalmometry compared to our study, the study by Sheik et al. showed slightly lower measurement results. A closer look at these studies revealed a lower resolution of the imaging procedures, which may have led to inaccuracies in the measurements. In addition, these studies were conducted internationally in different countries, meaning that ethnic origin may also play a role in the mean values of posterior exophthalmometry. The study by Lee et al. was conducted in South Korea^20^. In the Asian population, eye protrusion tends to be less pronounced^6^, yet the highest average values were found in comparison to other studies, at 11.1 mm^20^. Özgen et al.^22,23^ is the only previous study to date that has also worked with MR imaging data. Their study was carried out in Turkey and the eyeball also appears to lie on average deeper in the eye socket in their studies compared to ours^19^. This may therefore result in correspondingly higher measured values in posterior exophthalmometry. With regard to the slightly higher posterior exophthalmometry values in women in our study, a connection with hormone-dependent changes in orbital volume and the enlargement of posterior exophthalmometry can be discussed. The hormone-dependent reduction in orbital volume is reported primarily in women during and after menopause^24^. In addition, Japanese women and female members of the Aboriginal population were found to have a greater eye socket height^25^, suggesting that ethnic differences may also play a role. Further research should be conducted in this regard.

Another cross study difference is sample size, which is significantly larger in our study. Another point to consider is the study population. While our data was collected from a cross section of the general population, Lee et al. and Özgen et al. involved patients with corresponding disease processes in the eyes. Sex distribution is comparable between our studies and the ones discussed above. The main factor that could partly explain the different results, however, may be the fact that bulbar axial length was not taken into account in other studies. This is relevant since we know that this value influences protrusion of the eyes^5^. Our results were most consistent with the data from Sheik et al. from the Middle East. They also demonstrated a mean value of 7.1 mm in posterior exophthalmometry^21^. However, only 48 subjects with an average age of 35 years were included^21^. The advantage of posterior exophthalmometry is that it can always be performed and fewer artifacts appear on MRI. The anterior segment of the eye often exhibits impairments in image quality due to eyelid and eye movements, which makes it difficult to assign the correct landmarks for anterior measurements.

With respect to bulbar axial length, the statistical analysis shows that long bulbar lengths have an effect on both anterior and posterior exophthalmometry. This means that the eye or its center in the orbit does not change in comparison between a person with a longer bulbar axial length and a person with an average bulbar axial length. This is a new finding in our study. The correlation between the positional parameters and bulbar axial length illustrates the need to consider the results of exophthalmometry in light of bulbar axial length. In this case, the Ex/Axl ratio can be helpful for clinical evaluation, including the effect measurement after decompressive surgery for Grave´s disease. Since the study population in our cohort consists of a cross section of the general population, it can be assumed that the mean quotients of the reference group can serve as orientation and reference values—at least for patients with similar ethnic background.

Our study showed an association of the Ex/Axl ratio and the measured parameters with somatometric factors such as BMI, weight, waist and hip circumference (Table 3). Similar results have been reported by various studies worldwide. Schmidt et al. showed a positive correlation between BMI, waist and hip circumference and their MRI-measured exophthalmometry values^7^. Revankar et al. reported an association between BMI, height and weight and exophthalmometry measured by Hertel in an Indian cohort^26^. The MRI study by Erkoc et al. from Turkey also shows comparable results^27^. This indicates that somatometric parameters should be taken into account when interpreting exophthalmometry values and the Ex/Axl ratio. When analysing serum lipoproteins, our study found an association of Ex/Axl ratio with total cholesterol and triglycerides, an inverse association with HDL cholesterol and, in women, an association with LDL cholesterol. In the literature, cholesterol is mentioned as a possible risk factor almost exclusively in connection with Graves’ disease. In particular, total cholesterol and elevated LDL cholesterol are associated^28^, while an association with HDL cholesterol is only rarely reported^29^. The association between lipid metabolism and Graves’ disease is attributed to the inflammatory effect of serum lipids and the partially linked metabolic pathways with thyroid metabolism^28,29^. Other studies show no association between serum lipids and Grave’s disease^30^. In view of the fact that our cohorts show no associations with elevated TSH values and no Grave’s disease could be documented and proven, further investigations in the follow-ups of our cohorts are necessary. An association with HbA1c is also reported in relation to Grave’s disease, but the results are inconsistent. A connection via branches of metabolic pathways can also be suspected^31^.

The most significant limitation in our study is the exclusion of 549 subjects from the available and analyzable MRI images due to limited measurability. This creates the possibility of selection bias occurring. Overall, a standardized head position appears to be necessary for an adequate examination, possibly with fixation of the head during image acquisition to enable accurate interindividual and longitudinal intraindividual comparison of the measurement data. Furthermore, a strict forward gaze is important in order to avoid movement artifacts. Closure of the eyes should be avoided, as Bell’s phenomenon prevents the cornea and optic disc from lying in the same plane. Furthermore, an MRI is expensive compared to an exophthalmometer, so that this form of diagnosis will likely only be used to visualize the orbit and its structures in cases with an unclear cause of exophthalmos and/or inconclusive results on exophthalmometry and bulbar length measurements using other techniques like ultrasound or determination of the bulbar axial length with optical biometric devices, e.g. the IOL-master (Carls Zeiss Meditec, Jena, Germany).

## Summary

In summary of all results, there was evidence of clear differences in anterior exophthalmometry between men and women, so that cross-gender reference values do not appear useful. MRI images of the head were used to determine separate reference values for men and women, which may be useful in clinical practice. Furthermore, our study highlights the practical clinical significance of recording the individual bulbar axial length of the eyeball and anthropometric data for the personalized assessment of exophthalmos. The ratio (Ex/Axl) obtained from the measurement of anterior exophthalmometry and bulbar axial length suggest a method of distinguishing between pseudoexophthalmos and pathological, disease-associated protrusion of the eyeball (clinically manifest exophthalmos). This should be investigated further in clinical practice, for example in the quantification of the effect of decompressive orbit surgery in Grave´s disease.

## Data Availability

The data that support the findings of this study are available from Institute of Community Medicine, Study of Health in Pomerania, University Medicine Greifswald but restrictions apply to the availability of these data, which were used under license for the current study, and so are not publicly available. Data are however available from the authors upon reasonable request and with permission of Community Medicine, Study of Health in Pomerania, University Medicine Greifswald.
